# Human iPSCs and Genome Editing Technologies for Precision Cardiovascular Tissue Engineering

**DOI:** 10.3389/fcell.2021.639699

**Published:** 2021-06-28

**Authors:** Eric K. N. Gähwiler, Sarah E. Motta, Marcy Martin, Bramasta Nugraha, Simon P. Hoerstrup, Maximilian Y. Emmert

**Affiliations:** ^1^Institute for Regenerative Medicine (IREM), University of Zurich, Zurich, Switzerland; ^2^Wyss Zurich, University and ETH Zurich, Zurich, Switzerland; ^3^Division of Pediatric Cardiology, Department of Pediatrics, Stanford School of Medicine, Stanford, CA, United States; ^4^Vera Moulton Wall Center for Pulmonary Vascular Disease, Stanford School of Medicine, Stanford, CA, United States; ^5^Stanford Cardiovascular Institute, Stanford School of Medicine, Stanford, CA, United States; ^6^Molecular Parasitology Lab, Institute of Parasitology, University of Zurich, Zurich, Switzerland; ^7^Bioscience Cardiovascular, Research and Early Development, Cardiovascular, Renal and Metabolism, R&D BioPharmaceuticals, AstraZeneca, Gothenburg, Sweden; ^8^Department of Cardiovascular Surgery, Charité Universitätsmedizin Berlin, Berlin, Germany; ^9^Department of Cardiothoracic and Vascular Surgery, German Heart Center Berlin, Berlin, Germany

**Keywords:** human induced pluripotent stem cells (hiPSCs), CRISPR-Cas9, cardiovascular tissue engineering, regenerative medicine, cardiovascular disease modeling, 3D cell culture systems

## Abstract

Induced pluripotent stem cells (iPSCs) originate from the reprogramming of adult somatic cells using four Yamanaka transcription factors. Since their discovery, the stem cell (SC) field achieved significant milestones and opened several gateways in the area of disease modeling, drug discovery, and regenerative medicine. In parallel, the emergence of clustered regularly interspaced short palindromic repeats (CRISPR)-associated protein 9 (CRISPR-Cas9) revolutionized the field of genome engineering, allowing the generation of genetically modified cell lines and achieving a precise genome recombination or random insertions/deletions, usefully translated for wider applications. Cardiovascular diseases represent a constantly increasing societal concern, with limited understanding of the underlying cellular and molecular mechanisms. The ability of iPSCs to differentiate into multiple cell types combined with CRISPR-Cas9 technology could enable the systematic investigation of pathophysiological mechanisms or drug screening for potential therapeutics. Furthermore, these technologies can provide a cellular platform for cardiovascular tissue engineering (TE) approaches by modulating the expression or inhibition of targeted proteins, thereby creating the possibility to engineer new cell lines and/or fine-tune biomimetic scaffolds. This review will focus on the application of iPSCs, CRISPR-Cas9, and a combination thereof to the field of cardiovascular TE. In particular, the clinical translatability of such technologies will be discussed ranging from disease modeling to drug screening and TE applications.

## Introduction

### Historic Considerations: Stem Cell Research and the Foundation of Induced Pluripotent Stem Cells

**FIGURE 1:**
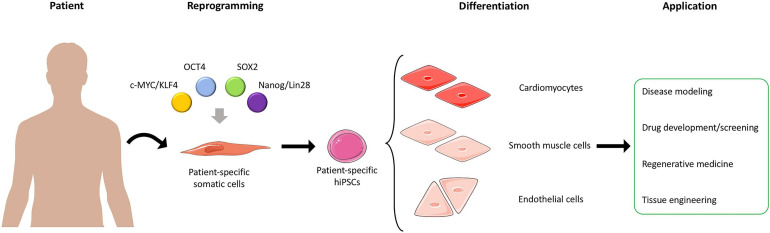
Generation and application of hiPSCs. Somatic cells are harvested from patients and reprogrammed into patient-specific hiPSCs. The hiPSCs can then be differentiated into different cell types, such as cardiomyocytes, smooth muscle cells, and endothelial cells, which can be used in different applications. Adapted from servier medical art, licensed under a Creative Commons Attribution 3.0 Unported License.

Stem cells (SCs) were first described in 1961 by Drs. James A. Till and Ernest A. McCulloch at Toronto University, where they discovered that mouse bone marrow-derived SCs possessed the unique ability to differentiate toward a multitude of different cell types (Till and McCulloch, 1961), thus laying the foundation for SC research. SCs are characterized by two properties: (i) self-renewal, which allow their indefinite division, producing unaltered daughter cells and (ii) the ability to exit self-renewal and differentiate into specialized cells giving rise to the three germ layers (i.e., ectoderm, mesoderm, and endoderm) (Wobus and Boheler, 2005). Naturally occurring SCs are classified by their self-renewal and differentiation potential: (i) totipotent SCs can differentiate into any cell type and can create an entire organism, the zygote is an example of totipotent cells; (ii) pluripotent SCs have the potential to differentiate into any cell type stemming from the cell lineages (ectoderm, mesoderm, and endoderm), human embryonic SCs (hESCs) is an example of pluripotency; (iii) multipotent SCs can differentiate into cells from a specific lineage, e.g., very small embryonic-like SCs (VSELs), which are early development SCs from adult tissues; (iv) oligopotent SCs can differentiate into a small number of cell types from a specific tissue, such as adult SCs; and (v) unipotent SCs, or progenitor cells, can differentiate into a single cell type (Zakrzewski et al., 2019; Liu et al., 2020). Artificially derived SCs, or human induced pluripotent SCs (hiPSCs), are reprogrammed from a terminally differentiated cell, but carry the same potency as hESCs. Additionally, nuclear transfer SCs (NTSCs), where the nucleus of a zygote is replaced with a somatic cell, are less effective than reprogrammed iPSCs (Zakrzewski et al., 2019; Liu et al., 2020). In 1962, the technique of somatic cell nuclear transfer (SCNT) provided the first evidence that terminally differentiated cells could reprogram into a pluripotent state (Gurdon, 1962; Wilmut et al., 1997). The year 1998 marked the discovery of the first hESCs, by James Thomson (Thomson et al., 1998), and the early 2000s demonstrated the fusion between hESCs and somatic cells that confirmed the potential to revert cells potency state to enable their reprogramming (Tada et al., 2001). iPSCs were first reported in 2006 by reprogramming innate adult somatic cells using four specific genes, octamer-binding transcription factor-3/4 (Oct3/4) and sex-determining region Y-box 2 (Sox2), combined to either c-Myc or kruppel-like factor-4 (Klf4), and homeobox protein nanog (Nanog) or lin-28 homolog A (Lin28) (Takahashi and Yamanaka, 2006; Okita et al., 2007; Takahashi et al., 2007; Yu et al., 2007; Figure 1).

### hiPSC Technology: Advantages and Disadvantages

The field of SCs considerably changed following the discovery of hiPSCs, and the emergence of reprogramming technology enabled the use of disease-specific hiPSCs, thereby circumventing the (ethical) limitations of hESCs (Wobus and Löser, 2011). In comparison with hESCs, the use of hiPSCs presents multiple advantages, such as reduced ethical requirements, high degree of dedifferentiation, high proliferation rate, and self-renewal ability (Wobus and Löser, 2011; Kumar et al., 2017). Such properties allow for the generation of libraries that can be used for the development of drug screening/response platforms, significantly reducing the related production costs (Wobus and Löser, 2011).

Additionally, allogenic hiPSCs can be reprogrammed from individual patients, thus retaining patient-specific properties, such as genetic information, and display no immunogenicity after transplantation (Guha et al., 2013). Hence, hiPSC-based therapies present a unique potential not only for disease modeling but also for precision medicine by establishing novel treatment strategies based on patient-specific phenotypes (Chun et al., 2011; Matsa et al., 2016). Furthermore, hiPSC’s capability to undergo almost indefinite proliferation cycles and the possibility to perform single cell clonal expansion make hiPSCs a reliable cell source for genome engineering approaches (Grobarczyk et al., 2015; Hotta and Yamanaka, 2015). Within the past decade, the application of these technologies has revolutionized several research areas, such as regenerative medicine, disease modeling, drug discovery, and human developmental biology demonstrating the reproducibility of this methodology (Kwon et al., 2018; Nikolić et al., 2018; Spitalieri et al., 2018; Cota-Coronado et al., 2019; Savoji et al., 2019; Figure 1).

Nevertheless, hiPSCs also present limitations related to the way they are produced. Former reprogramming approaches used retro- or lentiviruses as delivery system for transcription factors for somatic cell reprogramming arises safety concerns in regard to the integration of the viral system in the host genome, which can ultimately lead to genetic alteration, thus increasing tumorigenicity risks (Howe et al., 2008; Higuchi et al., 2015). More recent approaches aim at reducing the genetic alterations caused by reprogramming *via* non-integrating viruses (e.g., Sendai virus), episomal vectors, or through direct delivery of reprogramming factors, such as protein or mRNA to generate integration free hiPSCs (Kim et al., 2009; Okita et al., 2011; Diecke et al., 2015; Schlaeger et al., 2015; Rohani et al., 2016).

### hiPSC Technology: Regulatory Considerations and Clinical Application

As previously mentioned, the differentiation potential of hiPSCs and the numerous application possibilities of hiPSC-derived products are enormous; however, their clinical translation is still considerably hampered. Lack of scalable differentiation protocols, undifferentiated cell contaminates, as well as unknown *in vivo* hiPSC functionality and their potential to generate teratomas still limit the broader clinical application of such technology. To foster the use of hiPSCs and their derived products into the clinics, research groups are focusing on the establishment of reliable protocols for the isolation, generation, proliferation, and differentiation of hiPSCs following GMP-compliant regulations. In addition, preclinical efficacy and safety, ethical compliance, and respect of the regulatory guidelines need to be established *a priori* (Haake et al., 2019).

The rapid technology translation that hiPSCs are experiencing often reveals the gaps and limitations that still need to be faced, for example, genetic instability, immunogenicity, epigenetic abnormalities (Ben-David and Benvenisty, 2011; Laurent et al., 2011; Zhao et al., 2011; Liu et al., 2017; Bragança et al., 2019; Ratajczak, 2019), as well as publication bias and late translation into clinics after their generation, which can ultimately cause misuse of the patient genetic code (Wolinetz and Collins, 2020). In this regard, guidelines that protect the cell donors’ rights must be granted in order to protect future patients. This is of great concern as several clinical trials on hiPSCs are ongoing, some of which focusing on cardiovascular diseases (CVDs) (Deinsberger et al., 2020). Given the rapid propagation of such technology, the establishment of regulatory guidelines for disease modeling, drug discovery, and clinical translation is a must and has to be enlightened by the regulatory offices and SC societies.

### Rise of Genome Editing Technologies and CRISPR-Cas9

**FIGURE 2:**
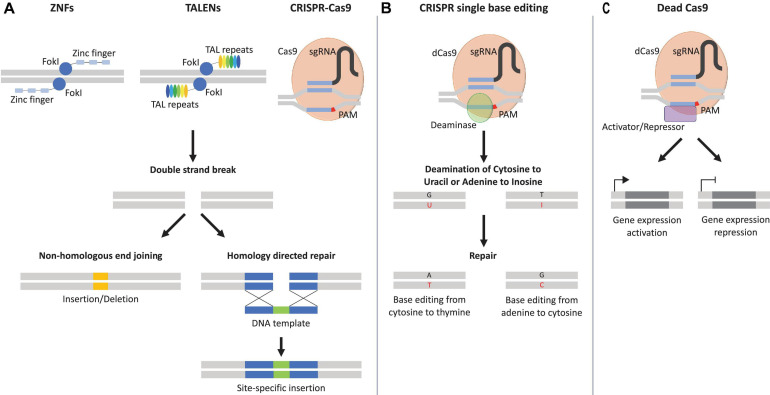
Genome editing tools. **(A)** Zinc finger nucleases (ZNFs) and transcription activator-like effector nucleases (TALENs) recognize specific genomic sites with their specific DNA-binding proteins, zinc finger and transcription activator-like repeats. Clustered regularly interspaced short palindromic repeats (CRISPR-Cas9) is directed to the specific genomic site with the help of the protospacer adjacent motif (PAM) in red and the sequence of the single guide RNA (sgRNA) in blue. Once the specific genomic site is recognized, the catalytical subunits, *Flavobacterium okeanokoites* type IIS restriction enzyme (*Fok*I), or CRISPR-associated protein 9 (Cas9) induces a double strand brake (DSB) and can be repaired either by non-homologous end joining that can lead to insertion/deletion or homology-directed repair with the help of DNA template that allows the introduction of specific mutations. **(B)** Catalytically inactive Cas9 or dead Cas9 (dCas9) fused to a deaminase (e.g., cytidine or adenine) can result in the conversion of cytosine–guanine base pairs to thymine–adenine or vice versa, without the need of a DSB. A = adenine, T = thymine, C = cytosine, G = guanine, U = uracil, I = inosine. **(C)** An additional variation of dCas9 fused to a transcriptional activator/repressor can induce transcriptional activation/repression of the targeted gene.

The idea of genomic information exchange *via* exogenous DNA homology recombination (HR) was initially demonstrated by Oliver Smithies (Smithies et al., 1985). This discovery was followed by the identification of meganucleases, which were able to introduce double strand breaks (DSBs) at specific sites in the genome, thus improving the insertion of exogenous DNA (Choulika et al., 1995; Cohen-Tannoudji et al., 1998). These findings opened the path for the development of the zinc finger nuclease (ZFN) technology in 2009, of transcription activator-like effector nuclease (TALEN) in 2011, and finally of clustered regularly interspaced short palindromic repeats (CRISPR) in 2013 (Geurts et al., 2009; Tesson et al., 2011; Gasiunas et al., 2012; Jinek et al., 2012; Cong et al., 2013; Mali et al., 2013b; Wang et al., 2013; Figure 2A). The CRISPR system originates from the defense mechanism found in archaea and bacteria (Terns and Terns, 2011). To be fully functional, the CRISPR system requires: (i) a DNA endonuclease, i.e., the CRISPR-associated protein 9 (Cas9), that cleaves the DNA specifically at the protospacer adjacent motif (PAM) and (ii) a small RNA molecule, known as the single guide RNA (sgRNA), that allows the CRISPR-Cas9 to target the specific genomic location and induce a DSB (Jinek et al., 2012; Anders et al., 2015; Figure 2A). The DSB triggers DNA repair *via* two different pathways, non-homologous end joining (NHEJ) or homology-directed repair (HDR) (Jinek et al., 2012; Gaj et al., 2013; Mali et al., 2013a; Figure 2A). NHEJ repair is a process that does not require a homology template and can thus introduce insertions or deletions (indel) at the cleavage site, thus causing a gene knockout if the indel occurs in an exon (Wyman and Kanaar, 2006; Hsu et al., 2014). To the contrary, HDR uses a homology DNA template to obtain high-fidelity repair, thereby allowing precise DNA insertions (Wyman and Kanaar, 2006; Hsu et al., 2014; Lin et al., 2014; Maruyama et al., 2015). In addition, further modifications of CRISPR-Cas9 allowed the development of single base editing, a fusion of cytidine or adenosine deaminase enzymes with Cas9, that enables single genetic modification without DSB (Komor et al., 2016; Porto et al., 2020). Within this technique, sgRNA targets the CRISPR-Cas9 base editor to the specific sequence of DNA. Subsequently, the cytidine deaminase induces the conversion of cytosine to uracil first, and then to thymine, with adenine as complementary base. On the other side, the adenine deaminase converts adenine into inosine, which is recognized as guanine, with cytosine as complementary base (Komor et al., 2016; Porto et al., 2020; Figure 2B). Besides the ability of CRISPR-Cas9 to permanently modify the genome, modifications of the catalytic site of the Cas9 nuclease allowed the generation of dead Cas9 (dCas9), which retains the specific binding ability to DNA, without inducing DSB (Qi et al., 2013; Hsu et al., 2014). Furthermore, fusion of dCas9 to transcription activators (e.g., VP16, VP64, p65) or transcription repressors (e.g., KRAB, SID) allowed to retarget CRISPR-Cas9 toward gene expression modulation (Dominguez et al., 2016; Mahas et al., 2018; Figure 2C). The CRISPR-Cas9 technology currently represents an established gene editing tool, which expands our understanding of genetic diseases by restoring genome integrity, and provided disease-specific cells for drug testing (Wang et al., 2014c; Zhang et al., 2014; Hinson et al., 2015).

### CRISPR-Cas9 Technology: Regulatory Considerations and Clinical Application

The enthusiasm around CRISPR-Cas9 technology has garnered a great degree of attention since its first reported use in 2013 (Musunuru, 2017b). However, the ethical (moral), biomedical, safety, and legal concerns regarding the use of such application to the medical (clinical) field are gaining importance (Brokowski and Adli, 2019). In 2015, the National Academics of Sciences, Engineering, and Medicine (NASEM) compiled one of the most extensive risk analysis reports on the use of such genome editing tools on humans (Brokowski and Adli, 2019). Finally, the committee agreed on somatic genome editing experimentation, but did not allow human genome modifications nor any kind of enhancement (Memi et al., 2018; Brokowski and Adli, 2019). In this regard, CRISPR-Cas9 technology is significantly helpful for the improvement of immunotherapies, organoid engineering, drug target identification, and disease-gene modifications (Singh et al., 2019). Particularly, this system offers the great potential to progress therapies against HIV, hemophilia, cancer, and any number of yet uncurable diseases (Singh et al., 2019).

## HiPSC Applications for Cardiovascular Research

The mammalian heart has limited regenerative capacity and is subjected to multiple genetic or non-genetic dysfunctions, thus resulting in heart diseases and/or failure (Skrzynia et al., 2014; Doppler et al., 2017). Currently, small and large animal models are used to study human heart diseases, yet inter-species differences as well as anatomical and physiological dissimilarities complicate the clinical translation of safe and effective therapies (Milan and MacRae, 2005; Camacho et al., 2016). Among the existing SC therapies, hiPSCs emerge as a potential cell source for CVD modeling and treatment (Park and Yoon, 2018; Sadahiro, 2019; Parrotta et al., 2020). Besides the possibility to generate hiPSCs from patients’ somatic cells and giving access to patient-specific cells, there is an added ability of hiPSCs to proliferate indefinitely, maintain the genetic information of their host, and differentiate into any cell type. This makes hiPSCs an ideal cell source to investigate CVD originating from acquired genetic or congenital defects thus establish a better understanding of the pathological mechanisms and molecular functions regulating cardiac disorders, thereby opening the path for the development of new diagnostic and therapeutic approaches (Matsa et al., 2016; Parrotta et al., 2020).

### hiPSC-Derived Cardiomyocytes

Over the years, several attempts have been made to establish hiPSC differentiation protocols that imitate the signaling pathways involved in embryonic cardiovascular development to obtain functionally mature cardiac cells (Skrzynia et al., 2014). Initially, differentiation of hiPSCs into cardiomyocytes (CMs) involved single cell suspension cultures. This induced hiPSCs to spontaneously aggregate and form embryoid bodies (EBs), thus mimicking embryogenesis (Maltsev et al., 1993; Yang et al., 2008). Following EB formation, cells differentiated into the three germ layers and finally acquired CM properties (Zhang et al., 2009; Zwi et al., 2009). However, despite the promising differentiation outcomes, the presence of serum in the medium made EB-based approaches prone to variability between samples, thus compromising the reproducibility level (Osafune et al., 2008). Subsequent improvements of EB-based differentiation protocols employed cytokines and growth factors, such as Wnt proteins, bone morphogenetic proteins (BMPs), activin A, and Notch signals, combined to their respective selective inhibitors (Chau et al., 2006; Laflamme et al., 2007; Wang et al., 2011; Lian et al., 2012; Burridge et al., 2014; Bastakoty et al., 2016; Abad et al., 2017), thus increasing the efficiency of differentiation. Simplified procedures were additionally established to eliminate the EB formation process by using a monolayer-based system (Paige et al., 2010; Zhang et al., 2012; Kadari et al., 2015; Talkhabi et al., 2016). Furthermore, the development of the two-step differentiation protocols enabled the derivation of cardiac progenitor cells (CPCs) from hiPSCs, followed by a second differentiation step where either CMs, smooth muscle cells (SMCs), or endothelial cells (ECs) could be obtained (Kattman et al., 2011; Cao et al., 2013).

However, the limitations for the differentiation approaches reported so far are that hiPSC-derived cardiac cells are heterogenous and lack cellular maturity, which subsequently result in fetal CM function and morphology (Veerman et al., 2015; Machiraju and Greenway, 2019). hiPSC-derived CM immaturity impairs the proper modeling of adult CVD due to the inability to fully reiterate aging-related disease phenotypes, as well as of genetic-related pathologies, thus negatively influencing their use for drug development and screening (Veerman et al., 2015; Goversen et al., 2018; Machiraju and Greenway, 2019). To overcome the lack of mature and homogenous hiPSC-CMs, new elaborate strategies were employed to include long-term culture periods, the use of hormones in the differentiation medium, mechanical or electrical stimulation, and the use of *in vivo* environments (Chan et al., 2013; Kamakura et al., 2013; Yang et al., 2014; Ruan et al., 2016; Cho et al., 2017; Kadota et al., 2017; Ebert et al., 2019; Machiraju and Greenway, 2019). These latest studies resulted in increased hiPSC-CM mitochondria generation, sarcomere maturation, change of energy source to fatty acid instead of glucose, as well as an enhanced electrophysiological metabolism and response electro-, mechanical-, or pharmacological stimulation (Chan et al., 2013; Kamakura et al., 2013; Yang et al., 2014; Ruan et al., 2016; Cho et al., 2017; Kadota et al., 2017; Ebert et al., 2019; Machiraju and Greenway, 2019).

### hiPSCs and CVD Modeling

An efficient reproduction of human diseases requires a relevant and precise model that recapitulates the pathophysiological mechanisms of the disease itself (Savoji et al., 2019). *In vitro* cell cultures and animal models can only recapitulate some physiological features of human diseases, but not the entire pathophysiological profile (Savoji et al., 2019). In fact, a broader application of existing *in vitro* disease models can be limited by the over simplification of *in vitro* approaches, the availability of patient-specific cells, and their limited proliferation potential, as in the case of CMs (Savoji et al., 2019). In this context, hiPSCs represent a promising cell source, owing to features such as human origin, unlimited proliferation capacities, and potential to differentiate into any cell type. Pioneering studies established hiPSCs from patients suffering from specific genetic conditions, thereby enabling the generation of disease models to mimic their particular molecular mechanisms (Park et al., 2008). Disease modeling based on hiPSCs generated great progress in the field of cardiovascular research, eventually providing the tools to acquire a more precise understanding of the underlying CVD mechanisms and to develop new therapeutic approaches. The ability of hiPSCs to maintain the genetic profile of the host while differentiating into cardiac-derived cells, such as CMs, SMCs, and vascular ECs, enabled the production of robust *in vitro* CVD models (Parrotta et al., 2020). Here, cardiac disorders including cardiomyopathies, channelopathies, and structural-based cardiac defects will be further discussed.

#### Cardiomyopathies

Cardiomyopathies encompass a number of disorders related to distinct genetic mutations, such as hypertrophic cardiomyopathy (HCM), dilated cardiomyopathy (DCM), arrhythmogenic right ventricular cardiomyopathy (ARVC), and left ventricular non-compaction (LVNC), and are defined by structural or functional dysfunction of the myocardium (Sisakian, 2014; Hannah-Shmouni et al., 2015).

HCM is one of the most common genetically inherited cardiomyopathies and is linked to ventricular and septum hypertrophy caused by hypertrophic CMs and fibrosis, resulting in decreased cardiac function (Wexler et al., 2009; Argulian et al., 2016). Hypertrophy of CMs is due to mutations in the genes responsible for sarcomere function, such as myosin heavy chains (MYH6, MYH7), myosin binding protein C (MYBPC3), troponin I (TNNT2, TNNT3), and tropomyosin-α-1 chain (TMP1) (Girolami et al., 2010; Sisakian, 2014). hiPSC technology allowed to better characterize HCM using patient-specific hiPSC-derived CMs harboring a MYH7 mutation (Lan et al., 2013; Dementyeva et al., 2019; Filippo Buono et al., 2020). A first study showed the potential of hiPSCs to mimic HCM phenotypes, such as enlarged cells, sarcomeric dysfunction, arrhythmia, and impaired calcium (Ca^2+^) handling, by using hiPSC-derived CMs from 10 patients carrying a missense mutation in the *MYH7* gene (A663H) (Lan et al., 2013). Findings on Ca^2+^ handling abnormalities allowed the identification of specific Ca^2+^-channel blockers (e.g., verapamil) and their function in restoring physiological Ca^2+^ regulation, hence averting HCM phenotype (Lan et al., 2013). An additional study on HCM using patient-specific hiPSC-derived CMs broadened our understanding of the underlying disease mechanisms and thereby enabled the development of new therapeutic approaches (Han et al., 2014). Particularly, the study used hiPSC-derived CMs harboring a missense mutation in the *MYH7* gene (R442G) in combination with genome-wide transcriptomics to investigate signaling pathways involved in the developmental process of CMs. This enabled the identification of potential therapeutic targets, such as Wnt, FGF, and Notch pathways (Han et al., 2014).

DCM represents one of the most common diagnoses in patients requiring heart transplantation and is associated with functional and structural impairment of the heart (Jacoby and McKenna, 2012). DCM relates to inherited gene mutations involved in sarcomeric protein synthesis [e.g., titin (TTN), MYH7, lamin A/C proteins (LMNA), desmin (DES)] or genes encoding ion channels [e.g., sodium channel protein type 5 subunit α (SCN5A)] (Gerull et al., 2002; Hershberger and Morales, 2007; Herman et al., 2012; Mcnally et al., 2013; Schultheiss et al., 2019). The use of patient-specific hiPSC-derived CMs again enabled to mimic the mutations causing the pathophysiological phenotype of DCM, thereby providing a better understanding of its underlying molecular and cellular mechanisms (Siu et al., 2012; Sun et al., 2012; Tse et al., 2013; Wang et al., 2014b; Hinson et al., 2015; Karakikes et al., 2015b; Lin et al., 2015; Wu et al., 2015; Wyles et al., 2016; Lee et al., 2017; Streckfuss-Bömeke et al., 2017; Yang et al., 2018; Shah et al., 2019).

Additionally, hiPSC technology allowed the modeling and the consequent discovery of specific therapeutic approaches for other cardiomyopathies, such as ARVC (Thiene et al., 2007; Caspi et al., 2013; Kim et al., 2013; Ma et al., 2013a) or LVNC (Kodo et al., 2016). In the case of ARVC, patient-specific hiPSC-derived CMs presenting the mutated *PKP2* gene allowed the establishment of an ARVC *in vitro* model by exposing hiPSC-derived CMs to induce metabolic aging conditions (Kim et al., 2013). A subsequent study showed that ARVC PKP2 mutation resulted in the upregulation of Wnt and PPAR-γ pathways and lipid accumulation, which allowed the identification of 6-bromoindirubin-3′-oxime (BIO) as an inhibitor of glycogen synthase kinase 3β and a potential treatment to reduce lipid accumulation (Caspi et al., 2013). LVNC was successfully modeled with hiPSC-derived CMs and showed abnormal signaling of transforming growth factor β (TGF-β) due to a mutation of cardiac transcription factor T-box protein 20 (TBX20), thus impairing proper compaction of CMs (Arbustini et al., 2016). Moreover, the correction of the mutation using CRISPR-Cas9 and inhibition of TGF-β allowed to restore normal phenotype (Kodo et al., 2016).

Overall, the implementation of hiPSC-derived CMs in the development of CVD-related models provided evidence of their ability to model complex cellular phenotypes and contributed to a better understanding of a number of involved mechanisms in the disease. Moreover, the knowledge obtained from hiPSC-based CVD models uncovered novel treatment approaches. Nevertheless, there are remaining questions regarding the heterogeneity and immaturity of the hiPSC-derived CMs that need to be further investigated to be able to utilize hiPSC technology to completely model CVDs.

#### Channelopathies

Channelopathies are the result of mutations in genes encoding ion channels and transporters and hence lead to a cardiac electrophysiology impairment (Abriel et al., 2015; Bezzina et al., 2015; Spears and Gollob, 2015). Disorders classified as channelopathies are congenital long QT syndrome (LQTS) and catecholaminergic polymorphic ventricular tachycardia (CPVT), which induce arrhythmias, ventricular fibrillation, and seizures ending with death (Abriel et al., 2015; Bezzina et al., 2015; Spears and Gollob, 2015). The introduction of hiPSC technology allowed for extensive studies into the underlying mechanisms of such ion channel-related disorders (Sala et al., 2019).

LQTS is characterized by a delay in the cell membrane repolarization after contraction and ventricular arrhythmias, ultimately leading to heart arrest, and it is the first and most studied arrhythmic syndrome using hiPSC-based models (Abrams et al., 2010; Sala et al., 2019). LQTS exists in more than 10 different subtypes, but research has mainly focused on LQTS1, LQTS2, and LQTS3, which result from a genetic mutation of the potassium voltage-gated channel subfamily Q member 1 (KCNQ1), subfamily H member 2 (KCNH2), and sodium voltage-gated channel α-subunit 5 (SCN5A), respectively (David et al., 2014). In-depth studies of such arrhythmic syndromes paved the way for the use of hiPSCs as well as for disease modeling (Moretti et al., 2010). Several studies carried out with patient-specific hiPSCs containing a mutation in the *KCNQ1*, *KCNH2*, or *SCN5A* gene displayed strong similarities in the derived CMs, such as prolonged action potential, reduced potassium or sodium currents, and subsequent impairment of the ion channel’s behavior (Itzhaki et al., 2011; Matsa et al., 2011; Egashira et al., 2012; Lahti et al., 2012; Bellin et al., 2013; Mehta et al., 2014; Wang et al., 2014c; Jouni et al., 2015; Ma et al., 2015). Further understanding of channelopathy molecular mechanisms facilitated by hiPSC-derived CMs showed that specific treatments using potassium and sodium ion channel inhibitors restore proper CM function (Fatima et al., 2013; Ma et al., 2013b; Terrenoire et al., 2013; Mehta et al., 2014; Malan et al., 2016).

CPVT comprises two subtypes; CPVT1 arises from mutations in the cardiac ryanodine receptor 2 (RYR2), and CPVT2 is caused by a mutation in the calsquestrin-2 (*CASQ2*) gene (Leenhardt et al., 2012; Roston et al., 2017). Both phenotypes result in tachyarrhythmias triggered by stress and exercise (Swan et al., 1999; Liu et al., 2013). RYR2 is responsible for the outflow of Ca^2+^ from the sarcoplasmic reticulum during depolarization, whereas CASQ2 proteins bind Ca^2+^ in the sarcoplasmic reticulum (Leenhardt et al., 2012; Roston et al., 2017). Multiple hiPSC-based models recapitulated CPVT phenotypes, as well as showed elevated diastolic Ca^2+^ concentrations, reduced Ca^2+^ in the sarcoplasmic reticulum, and increased arrhythmias (Jung et al., 2012; Kujala et al., 2012). Furthermore, the use of hiPSC-based models identified new therapeutics, such as dantrolene, β-blockers, and flecainide (Itzhaki et al., 2012; Jung et al., 2012; Preininger et al., 2016; Sasaki et al., 2016).

Collectively, the introduction of hiPSC-derived CMs in the modeling of channelopathies allowed for the acquisition of a new knowledge into the mechanisms of cardiovascular electrophysiology. This further established novel therapeutic approaches to attenuate the observed pathophysiological conditions (Swan et al., 1999; Liu et al., 2013; Roston et al., 2017). However, the strength of the clinical translation of such technology is limited due to the lack of complete maturity of hiPSC-derived CMs, which still remains an issue that needs further investigation.

#### Structural Defects

Structural heart diseases typically feature an abnormality in the structure of the heart, valves, and/or vasculature and represent a rapidly growing CVD area that has been successfully modeled using hiPSC technology (Peng et al., 2019). In particular, hiPSC-derived models for bicuspid valvular and aortic calcification identified mutations in the notch homolog 1 (*NOTCH1*) gene (Garg et al., 2005; Theodoris et al., 2015). Additionally, the generation of hiPSC-derived SMCs allowed to recapitulate the multiple features of supravalvular aortic stenosis (SVAS) including mutations in the elastin (*ELN*) gene (Ge et al., 2012; Kinnear et al., 2020). Thus, hiPSC-derived models provide a great tool to investigate cellular function as well as the molecular mechanisms behind such diseases, ultimately leading to new therapeutic approaches (Ge et al., 2012; Chailangkarn and Muotri, 2017; Kinnear et al., 2020).

## HiPSCs for Tissue Engineering Applications

### Regenerative Medicine

hiPSC-based technology accelerated drug development and their safety evaluation by providing tools to investigate various disease mechanisms and to screen for potential treatments. Although the current therapeutic approaches provide a treatment option for specific diseases, they do not induce regeneration of the damaged cardiac tissues to this date (Altara et al., 2016). Therefore, the discovery of patient-specific hiPSCs offered a potential novel treatment option to replace damaged cardiac tissue and opened a new chapter in the field of regenerative medicine (Menasché, 2018; Wang et al., 2018; Paik et al., 2020). Furthermore, owing to the ability of potentially differentiating into any desired cell type, hiPSCs provide a means to patient-specific cardiac tissue regeneration, thus facilitating allogeneic transplantation (Šarić et al., 2008; Zhang et al., 2009; Narsinh et al., 2011; Kishino et al., 2020). The first studies using hiPSC-derived CMs to restore cardiac diseased tissue involved the direct injection of cells in the damaged area (Nelson et al., 2009; Ye et al., 2014; Shiba et al., 2016; Liu et al., 2018). However, despite the promising initial results, the implementation of hiPSC technology into the clinic faces multiple challenges (Ma et al., 2011). One of the main limitations is due to the hiPSC-derived CM purity and the risk of teratoma formation post-transplantation caused by the presence of undifferentiated cells (Ma et al., 2011). Nowadays, several methods are in place to overcome this lack of cell purity, such as the use of lactose instead of glucose supplemented medium to eliminate undifferentiated cells (Ma et al., 2011; Zhang et al., 2012). As already mentioned, another limitation is the immature phenotype of hiPSC-derived CMs (Lundy et al., 2013; Machiraju and Greenway, 2019), as well as the low engraftment efficiency of the implanted CMs after single cell transplantation (Lemcke et al., 2018; Park et al., 2019). Therefore, cardiovascular regenerative medicine shifted from single cell injections of hiPSC-derived CMs toward alternative approaches to improve engraftment. By using tissue engineering (TE) approaches, such as cell aggregates or patches, cell retention and engraftment efficiency were greatly improved (Masumoto et al., 2012; Emmert et al., 2013a, b; Wendel et al., 2015). This led the field of cardiovascular regenerative medicine toward the implementation of three-dimensional (3D)-based culture systems.

### 3D Cell Culture System

A major step toward the generation of cardiovascular tissue is to recapitulate the molecular environment, which is best accomplished by using 3D culture systems, such as scaffold-based, organoids, and organs-on-a-chip technologies (Zimmermann et al., 2002a, 2004; Clevers, 2016; Günter et al., 2016; Rosales and Anseth, 2016; Madl et al., 2018; Ronaldson-Bouchard and Vunjak-Novakovic, 2018). While two-dimensional culture systems are the standard approach in cardiovascular research, these fail to recapitulate the complex cellular composition and extracellular interactions existing in native tissues. In order to properly mimic cardiac function, it is therefore important to consider the 3D tissue composition to re-create the cardiovascular cellular and extracellular environments. The use of 3D culture systems presents the ability to closely mimic the *in vivo* structure, microenvironment, cell–cell interaction, and cell–extracellular matrix (ECM) interaction, making it an interesting technology for disease modeling, drug development/screening, and TE applications (Fong et al., 2016; Correia et al., 2018; Chaicharoenaudomrung et al., 2019). Different 3D culturing approaches are available, including non-scaffold-based systems (e.g., spheroids and organoids) and scaffold-based systems (e.g., tissue-engineered constructs), that recapitulate ECM features.

#### hiPSCs in Cardiac Spheroids

Cardiac spheroids are typically used to mimic the native 3D cellular environment by including multiple cell types in a self-assembly process (Polonchuk et al., 2017; Hoang et al., 2018; Yan et al., 2019). In this context, the number of cells used to generate the spheroids can have an impact on the viability of cells, especially the ones forming the core, due to reduced oxygen supply when the spheroids diameter exceeds beyond diffusion barrier limit (Tan et al., 2017). Different studies generated spheroids by combining hiPSC-derived CMs with structural heart cells, such as cardiac fibroblasts (CFs), in order to closely recapitulate the native microenvironment of the myocardium (Polonchuk et al., 2017; Tan et al., 2017; Hoang et al., 2018; Mattapally et al., 2018; Yan et al., 2019). A recent study generated cardiac spheroids by co-culturing hiPSC-derived CMs with CFs and cardiac ECs and exposed them to various Food and Drug Administration (FDA)-approved drugs to test their potential as platform for cardiotoxicity assay (Archer et al., 2018). A different study produced cardiac spheroids, representative of the morphology and biochemistry of myocardial tissue out of hiPSC-derived CMs, hiPSC-derived CFs, and cardiac ECs (Polonchuk et al., 2017). Furthermore, the hiPSC-based cardiac spheroids allowed to investigate the underlying cardiotoxicity mechanisms of doxorubicin (Polonchuk et al., 2017). Another relevant study used cardiac spheroids composed of CMs and mesenchymal stem cells (MSCs) derived from hESCs as an *in vitro* platform to model fibrosis (Lee M.O. et al., 2019). Treatment of the cardiac spheroids with TGF-β induced a fibrotic phenotype (Lee M.O. et al., 2019).

#### hiPSCs in Cardiac Organoids

Similar to spheroids, organoid culture systems are described as a 3D approach that includes a specific cellular organization and a precise architecture that ultimately relies on a process of self-assembly (Lancaster and Knoblich, 2014; Chaicharoenaudomrung et al., 2019; Hofbauer et al., 2020). Since the first *in vitro* creation of the murine small intestinal organoid (Sato et al., 2009), many fields have been using organoid-based culture systems to mimic and recapitulate organ-like tissue architecture and cellular composition (Sato et al., 2009; Huch et al., 2013; Lancaster et al., 2013; Chen et al., 2017). The development of hiPSC technology opened the path for the development of patient-specific hiPSC-based organoids required to re-create functional cardiac organoids (Mills et al., 2017; Richards et al., 2017). Other studies aimed at producing 3D vascular networks organoids by means of an *in vitro* co-culture of hiPSC-derived ECs with vascular cells, such as pericytes (Kusuma et al., 2013; Orlova et al., 2014; Chan et al., 2015). A more recent study generated organoids that recapitulated blood vessels, by differentiating hiPSCs in suspension into the mesodermal lineage prior to inducing EC differentiation. The resulting blood vessel organoid displayed morphological and functional similarities with native human blood vessels when implanted in the kidney capsule of mice (Wimmer et al., 2019). However, the self-assembly process used in most organoid procedures is still one of the limiting factors for a consistent generation of cardiovascular tissues. Specifically, this process is not yet defined, but is rather a random method resulting into heterogenous organoids in regard of cell composition, size, and shape (Brassard and Lutolf, 2019). Particularly, the size of the organoids is limited due to the manufacturing approach, thus impairing the application of organoids to regenerative medicine (Brassard and Lutolf, 2019). Nonetheless, the application of hiPSC-derived cardiac organoids to disease modeling presents multiple advantages in precision medicine by allowing the simultaneous study of a large variety of phenotypes, but also a robust technology applicable in drug development and screening (Mills et al., 2017; Voges et al., 2017; Hoang et al., 2018; Nugraha et al., 2018; Mills et al., 2019; Nugraha et al., 2019; Filippo Buono et al., 2020).

#### hiPSCs for Cardiovascular Tissue-Engineered Constructs

TE aims to recreate functionally native tissue by recapitulating the exact cellular composition and ECM structure by means of bioengineering methodologies. The final purpose is to replace a diseased tissue or organ and/or develop and test new therapeutics. The application of TE to the medical field is of high clinical relevance for the regeneration of tissues with limited self-regenerative potential, such as the heart, pancreas, bone, and cartilage (Risbud et al., 2001; Sepulveda et al., 2002; Niknamasl et al., 2014; Emmert et al., 2017).

The classical TE approach relies on the combination of cells and biocompatible scaffolds to engineer tissue constructs with similar properties to native tissues. In this context, the scaffold not only is responsible for the structural support of the seeded cells but also can impact different functional aspects, such as cell survival, proliferation, and differentiation (Vunjak-Novakovic et al., 2011; Buikema et al., 2013; Chun et al., 2015). The first engineered heart tissue was developed using rat CMs cultured on a scaffold (Zimmermann et al., 2002b). This pioneered the development of novel approaches using various synthetic (e.g., polylactide, polyglycolide, lactide and glycolide copolymer, polycaprolactone, and polyisopropylacrylamide) and/or natural (e.g., collagen, cellulose, chitosan, hyaluronic acid, silk fibroin, and decellularized native tissue) (Vunjak-Novakovic et al., 2011; Buikema et al., 2013; Chun et al., 2015) scaffolds in the pursuit to optimize cardiovascular tissue-engineered constructs (Murry et al., 1996; Planat-Benard et al., 2004; Ravichandran et al., 2013; Wendel et al., 2014). The advent of PSCs, more specifically hiPSCs, offered access to an unlimited source of autologous cells with the ability to theoretically differentiate into any cell type in the body. Furthermore, the combined use of a scaffold, hiPSCs, and their metabolites provided the ability to generate personalized scaffolds, thus creating a tool with tremendous application potential (Noor et al., 2019). The first engineered cardiovascular tissue construct was based on the differentiation of ESCs into CMs and displayed the potential of PSCs to recapitulate metabolic and mechanical functions of the native myocardium (Stevens et al., 2009).

One fundamental aspect of tissue-engineered constructs, which strongly impact their *in vivo* behavior once implanted, is their fabrication method. While cardiac patches are generated by stacking cell monolayers to produce a functional tissue, 3D cardiac tissues make use of scaffolds to optimize cell proliferation, differentiation, and survival and mimic myocardium ECM structure and composition (Eschenhagen et al., 1997; Sawa et al., 2012). Various studies produced cardiac tissues using the cell sheet approach with hiPSC-derived cardiac cell types and induced *in vivo* recovery of damaged heart tissue (Masumoto et al., 2014; Masumoto et al., 2016; Ishigami et al., 2018). Nonetheless, the number of layers comprising the cardiac cell sheet has to be limited to maximize oxygen and nutrients diffusion and avoid tissue necrosis, which was improved using a biodegradable biomaterial to facilitate oxygen and nutrient transfer (Matsuo et al., 2015). In the same research framework, the combination of 3D scaffolds and hiPSC technology was explored to generate cardiac tissue constructs and establish new approaches to treat cardiovascular-related defects (e.g., vascular grafts and heart valves). In this context, TE approaches were combined with hiPSC-derived ECs and hiPSC-derived SMCs to produce vessels and/or valvular constructs, which recapitulated the physiological features of their native counterparts (Nakayama et al., 2015). A recent study used hiPSC-derived vascular SMCs cultured on polyglycolic acid (PGA) scaffold to manufacture vessel substitutes, which presented similar mechanical resistance as the clinically used prosthesis (Luo et al., 2020).

Valvular heart disease (VHD) is another type of cardiac defect that may benefit from hiPSC technology. VHD remains as one of the major heart problems and requires replacement to restore proper valvular function in the majority of patients (Kheradvar et al., 2015). The currently available prostheses are either mechanical or bioprosthetic valves (Poulis et al., 2020), and although they represent the current standard of care, both substitutes still present with significant limitations (e.g., degeneration, thromboembolic risk, need for anticoagulation treatment) as reviewed elsewhere (Head et al., 2017; Fioretta et al., 2020b; Poulis et al., 2020). Particularly, TE approaches could provide a potential solution to overcome the current limitations by generating tissue-engineered heart valves (TEHVs) with the ability to grow and remodel within the patient (Schmidt and Hoerstrup, 2006; Emmert and Hoerstrup, 2017). In this context, multiple cell and SC sources have been investigated for the generation of ready-available regenerative TEHVs (Cambria et al., 2017; Emmert, 2017). At the dawn of this technology, autologous cells and tissue sources were cultured in a bioreactor system to favor cell proliferation and ECM production (Weber et al., 2012b; Fioretta et al., 2018). However, the technical and logistical challenges of this TE approach (i.e., cell isolation and expansion, donor-to-donor variability, and unknown *in vivo* remodeling outcomes) led to the implementation of one-step interventions (Emmert et al., 2017). In this context, bioresorbable polymer-based TEHVs were adopted preclinically in combination with pre-seeding procedures using autologous SCs [bone marrow mononuclear cells (BMMCs) and fetal cells] to modulate the early *in vivo* inflammatory response (Emmert et al., 2011, 2012, 2014; Weber et al., 2011, 2012a; Fioretta et al., 2020a). However, further studies need to clarify the remodeling effects that SCs can develop in combination to the valvular hemodynamic conditions in order to exclude potential deleterious effects on the implanted substitute as well as guarantee long-term functionality and adaptive remodeling (Fioretta et al., 2020a; Mela, 2020). Akin to the use of hiPSC-derived SMCs for the generation of vascular grafts, the use of hiPSCs derivatives could be of high potential in the production of autologous TEHV.

Moreover, the combination of natural or synthetic scaffolds with hiPSC technology for TE constructs demonstrated that along with providing proper cell adhesion and proliferation, scaffold vascularization was triggered and favored tissue remodeling of the TE construct once implanted (Gaballa et al., 2006; Miyagi et al., 2011; Shi et al., 2011; Geng et al., 2018; Lee A. et al., 2019). Particularly, proven the promising preclinical and clinical outcomes of decellularized tissue-engineered matrices (Schmidt et al., 2010; Driessen-Mol et al., 2014; Emmert et al., 2018; Lintas et al., 2018; Motta et al., 2018, 2019, 2020), such starting materials could provide an interesting substrate to be implemented within the hiPSCs therapies options.

## Drug Development and Screening

The preclinical development of new therapeutics or drugs involves multiple processes, such as drug screening, *in vitro* and *in vivo* pharmacological and pharmacokinetic activity assessments, and safety analysis (Gromo et al., 2014). The discovery of hiPSCs provided a new platform that significantly changed preclinical drug screening and development (Paik et al., 2020). The combination of hiPSCs to next-generation sequencing, genome-wide association studies, and libraries of molecules allowed for the establishment of a powerful cell-based platform, which enabled the investigation of potential therapeutic molecules (del Álamo et al., 2016; Sharma et al., 2018; Paik et al., 2020). The application of an hiPSC-based platform to cardiovascular pharmacology facilitated the generation of patient-specific and disease-specific cell sources, such as hiPSC-derived CMs, that exhibited pathophysiological phenotypes similar to the one observed in diseased patients, thus providing a screening platform for existing and new drugs (Chun et al., 2011; del Álamo et al., 2016; Matsa et al., 2016; Sharma et al., 2018). In particular, the use of hiPSC-derived CMs as a screening tool to investigate the safety of drugs used in the treatment of HCM (such as metoprolol and verapamil) was successfully proven (Han et al., 2014). Furthermore, patient-specific hiPSC-derived CMs were employed to assess the cardiotoxicity of chemotherapeutics, such as doxorubicin and trastuzumab, showing how prolonged exposure to such drugs, induced decreased cell viability, perturbation in Ca^2+^ management, mitochondrial malfunction, and contraction impairment (Burridge et al., 2016; Chaudhari et al., 2016; Kitani et al., 2019). The available high-throughput assays and high-scale production of hiPSC-derived CMs enabled the simultaneous screening of multiple drugs on different lines of hiPSC-derived CMs, thus generating a faster assessment of drug-induced cardiotoxicity (del Álamo et al., 2016; Denning et al., 2016; Blinova et al., 2018; Grimm et al., 2018; Millard et al., 2018; Sharma et al., 2018; Burnett et al., 2019). Drug cardiotoxicity represents one of the main concerns in cancer treatment; thus, the development of hiPSC-based therapies provided an unprecedented advantage to evaluate and discover the cardiovascular toxicity of specific drugs, prior to clinical trials (Sharma et al., 2018).

Although 2D *in vitro* cell models are routinely used, such technologies lack the structural complexity, electrophysiology, and expression profile of human tissues, which can reduce the fidelity of the model and impair accurate characterization of drug effects and toxicity predictions on cells (Veerman et al., 2015; Machiraju and Greenway, 2019). In fact, the cell types constituting human organs are co-dependent for the exchange of molecules promoting growth, cell–cell interaction, and cell–ECM interaction (Paschos et al., 2015). The advent of 3D cell culture systems allowed to generate a more faithful representation of the cardiac cellular microenvironment, thus overcoming the limitations of 2D culture systems (Fong et al., 2016; Correia et al., 2018; Ronaldson-Bouchard et al., 2018). It has been suggested that the presence of 3D architecture and ECM influences the drug diffusion and dose-dependent toxicity, thereby providing a more reliable readout than 2D screening systems (Langhans, 2018; Zuppinger, 2019). The implementation of TE approaches further promoted a transition from 2D to 3D models in the drug screening processes. In this regard, several achievements have been made in the development of hiPSC-based tissue-engineered 3D cardiac platforms (Langhans, 2018; Zuppinger, 2019). As an example, hiPSC-derived CMs were used to manufacture multilayered 3D cardiac tissues and adopted to characterize drug-induced cardiotoxicity of various known drugs, such as doxorubicin, hERG-type potassium channel blockers, and isoproterenol (Takeda et al., 2018). Other approaches involve 3D printing of micro-physiological platforms simulating heart tissue, which were implemented in drug studies (Polini et al., 2014; Lind et al., 2017). To be noticed, a recent study developed an organoid-based platform and established a method to investigate drug-induced cardiotoxicity at the tissue level (Richards et al., 2020).

## HiPSCs and Genome Editing Technologies for Cardiovascular Applications

The study of site-specific nucleases (SSNs) started with the findings that DNA DSBs were repaired by the cell repair machinery using the HDR or NHEJ pathway (Rouet et al., 1994a, b). Subsequently, SSNs were implemented as a tool to engineer the genome at targeted sites. The initial nucleases were found to be hybrid proteins, known as ZFNs and TALENs, and were followed by the latest, CRISPR-Cas9 (Musunuru, 2013). The discovery of these nucleases revolutionized the field of genome engineering and biomedicine (Musunuru, 2013). While ZFNs and TALENs specific targeting relies on a protein-based system with customized specificity to DNA, CRISPR-Cas9 specific DNA targeting is RNA-guided and relies on a gRNA of 20 nucleotides (Corrigan-Curay et al., 2015). CRISPR-Cas9 has quickly become the most used gene editing technology due to the simplicity and adaptability of the RNA-based targeting system, which is easily customized to target any wanted sequence in the genome (Sander and Joung, 2014; Corrigan-Curay et al., 2015). These molecular editing tools have the ability to induce a DSB at a desired location in the genome, thus leading to either NHEJ or HDR for DNA repair. This allows for the introduction of a targeted mutation related to a diseased phenotype or to correct a disease-causing mutation (Cong et al., 2013). Progress in genome engineering methods, especially using CRISPR-Cas9, led the way for the development of isogenic cell lines, which in turn allowed for the introduction or correction of a desired mutation and, thus, the generation of several disease models (Hockemeyer and Jaenisch, 2016). Furthermore, the combination of hiPSC technology and the improvement of hiPSC differentiation protocols with CRISPR-Cas9 gene editing tools have established powerful approaches for SC-related research, human disease modeling, and drug development/screening (Schwank et al., 2013; Johnson and Hockemeyer, 2015; Matano et al., 2015; Musunuru, 2017a).

Off-target effects are a limitation in the use of CRISPR-Cas9 technology and other gene editing tools (De Masi et al., 2020). Off-target effects arise from unspecific targeting of the Cas9 nuclease, due to non-specific binding of the designed sgRNA sequence (Fu et al., 2013; Tycko et al., 2016; Naeem et al., 2020). The design of sgRNA is therefore very important to properly guide the Cas9 nuclease and aims to the minimization of unspecific genome binding (off-target events). Several studies assessed the off-target occurrence in specific cell lines showing that the frequency is cell-dependent (Fu et al., 2013), and that iPSCs have a low off-target occurrence (Smith et al., 2014; Veres et al., 2014). Nevertheless, off-target effects still represent a limitation that requires further investigation to broaden the clinical use of such gene editing tools (Rincon et al., 2015; Naeem et al., 2020). An additional constraint that can affect gene editing efficiency is linked to the delivery system (e.g., electroporation, micro-injection, transfection, lipofection, or viral vector) and the format of the Cas9 nuclease components [e.g., plasmid, mRNAs, or ribonucleoprotein complex (RNP)] (Lino et al., 2018). Depending on the format of Cas9, cells and/or tissues are subjected to a prolonged exposure; thus, transient expression of the Cas9 using RNP format is preferable to limit off-target effects and avoid unwanted gene editing (Moore et al., 2015; Liang et al., 2017; Sakuma et al., 2018; Singh et al., 2018).

The implementation of gene editing technology to CVD modeling using hiPSCs produced isogenic hiPSCs, thus creating genetically matched cells containing only selected inserted mutations. This system could correlate specific mutations to the observed phenotype (Dzilic et al., 2018) as well as generate hiPSC-based disease models that recapitulated CVD (Wang et al., 2014a; Kodo et al., 2016; de la Roche et al., 2019; Garg et al., 2019; Mcdermott-roe et al., 2019). Besides introducing specific mutations, various studies showed the potential of CRISPR-Cas9 in the correction of single genetic mutations related to various diseases, such as HCM, DCM, and LQTS (Karakikes et al., 2015b; Limpitikul et al., 2017; Seeger et al., 2019). In addition to gene editing, the CRISPR-Cas9 system also possesses the ability to regulate gene expression. The development of a catalytically inactive form of the Cas9 nuclease, known as dCas9, repurposed Cas9 into a protein able to specifically bind DNA and interfere with the gene expression, when targeted to a promoter or a regulatory sequence using a gRNA (Gilbert et al., 2013; Qi et al., 2013). Studies showed that the combination of the dCas9 with a transcriptional repressor and the specific targeting of the dCas9 toward a promoter or a regulatory sequence induced a downregulation of the targeted gene (Gilbert et al., 2013; Mandegar et al., 2016). Alternatively, studies demonstrated that coupling of dCas9 to a transcriptional activator induced the recruitment of gene effectors, thus leading to an increased expression of the gene in question (Maeder et al., 2013).

Moreover, studies showed the ability of gene editing tools to repurpose terminally differentiated cells, without having to go through a pluripotent state (Gao et al., 2013). Recent investigations showed the possibility of direct reprogramming of terminally differentiated fibroblasts into skeletal myocytes by targeting dCas9 coupled to an activator toward the *Myod1* gene, inducing high expression of myogenic markers, thus promoting differentiation from fibroblast directly into skeletal myocytes (Chakraborty et al., 2014). This approach could directly generate CMs and other relevant cardiac lineages *in vivo*, by reprogramming resident CFs to restore and regenerate damage tissue after injury.

The advances in gene editing and hiPSCs technologies triggered the research of novel therapeutic approaches including the possibility to either correct or introduce genetic mutations in patient-specific hiPSCs (Hockemeyer and Jaenisch, 2016). Furthermore, the pluripotency of hiPSCs provided massive potential to differentiate edited hiPSCs into any required cell type (e.g., CMs or cardiovascular-related cells). These autologous cells can then be transplanted back into the patient, thus circumventing the immunological response (Kishino et al., 2020). As previously mentioned, the transplantation and viability efficiency of single cells is highly dependent on the cells engraftment into the damaged tissue, which in the case of the heart has been proven very low (Lemcke et al., 2018; Park et al., 2019). In many cases, the host tissue requires the engraftment of a high number of corrected cells to overcome the diseased area. Advances in TE provided the necessary tools to overcome the limitations of single cell transplantation allowing the generation of bioengineered constructs, such as cardiac patches, vascular grafts, and heart valves (Matsuo et al., 2015; Nakayama et al., 2015; Fioretta et al., 2018; Lee A. et al., 2019; Luo et al., 2020). In this context, patient-specific somatic cells can be minimally invasively harvested, reprogrammed into hiPSCs, gene edited to correct disease-causing mutations, and then re-differentiated into the required cell types to produce autologous tissue constructs that could be implanted into the patient without stimulating an immune response. Furthermore, the CRISPR-Cas9 system has shown the ability to manipulate the immunogenicity of hiPSC-based tissue constructs by inducing the expression of immune suppressive molecules, thus reducing rejection by the host immune system (He et al., 2017; Zhao et al., 2020). Specifically, CRISPR-Cas9 has been used to generate hiPSCs lacking the human leukocyte antigen (HLA), thus reducing their immunogenicity (Han et al., 2019; Jang et al., 2019; Zhao et al., 2020).

While still in its infancy, the avenue of gene editing technology provides new opportunities to tackle the many challenges of disease modeling, regenerative medicine, and TE. Genome editing completely changed the landscape of cardiovascular research and has been demonstrated to be a powerful tool to study and manipulate genome-related molecular function.

## Discussion

**FIGURE 3:**
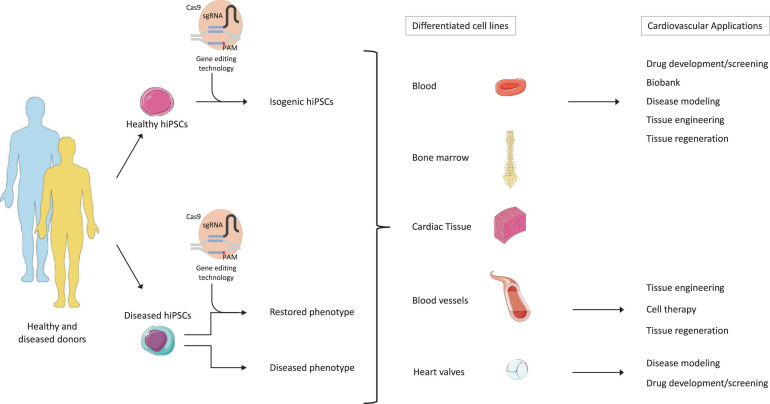
Therapeutic potential of hiPSC technology combined to gene editing and tissue engineering. The figure describes the potential applications of hiPSCs and gene editing. First, hiPSCs would be generated from the reprogramming of patient-specific healthy or diseased somatic cells. Second, gene editing tools, such as CRISPR-Cas9, would then generate isogenic cell lines harboring specific genetic mutations in the healthy hiPSCs, but also correct disease-causing mutations in the patient populations. The generated hiPSCs could then be re-differentiated into various cell types and/or tissue. Finally, the differentiated isogenic hiPSCs could be implemented in drug development/screening processes, biobanking, disease modeling, and tissue engineering. On the other side, the differentiated diseased hiPSCs could be further employed for disease modeling and drug development/screening. When genetic mutations are corrected, then cell-based therapy and tissue regeneration purposes can be applied. Adapted from servier medical art, licensed under a Creative Commons Attribution 3.0 Unported License.

Therapeutic approaches to treat CVDs and regenerate severely impaired tissues are under continuous development. However, to date, except heart transplantation for advanced heart failure, no curative treatments are available. The discovery of hiPSCs has provided the researchers a novel tool to investigate the underlying mechanisms of human diseases, including CVD. The high proliferative capacity and the ability to differentiate into any cardiac cell type opened the path for the generation of *in vitro* disease models, recapitulating the biomolecular and structural pathologies arising from cell mutations. The parallel advances of genome engineering technologies, such as CRISPR-Cas9, further optimized disease modeling processes using hiPSCs. Furthermore, the combination of hiPSCs and CRISPR-Cas9 technologies gave a new perspective for personalized medicine, by providing the necessary tools to correlate the disease phenotype with the underlying environmental, genetic, and molecular mechanisms for each individual patient (Hsu et al., 2014; Karakikes et al., 2015a). Indeed, several studies demonstrate that not only the influence of the patient’s genetic profile but also the environmental exposure affects the development of a disease and its outcome. In other words, each human being presents discrepancies in disease initiation and progression, reinforcing the importance of personalized medicine (Loscalzo and Handy, 2014; Smith and White, 2014). In this context, hiPSCs represent a promising cell source, giving access to patient-specific hiPSC-derived cardiac cells that retain the genetic background and the environmental influence of the patient they originate from, thus allowing to monitor and recapitulate the patient’s phenotype and their response to drugs (Engle and Puppala, 2013; Liang et al., 2013; Figure 3). Furthermore, CRISPR-Cas9 technology brings another tool to investigate the impact of genetic variation against the environmental influence, by creating isogenic hiPSC lines harboring a specific mutation out of healthy donor hiPSCs and comparing the resulting phenotype with the one of hiPSCs reprogrammed from diseased patients (Hsu et al., 2014; Dzilic et al., 2018). In addition, the combination of hiPSC technology with organoid/3D cell culture systems was used to generate biobanks, which could be used in various contexts, such as drug discovery, and as proof-of-concept for genetic disease correction in combination with CRISPR-Cas9, before proceeding to clinical trials (Geurts et al., 2020; Figure 3).

To properly study CVD origins and their 3D environment, future treatment strategies should implement co-culture systems of hiPSC-derived CMs with other cardiac cell types and TE approaches to closely mimic *in vivo* pathologies. Such approaches would recapitulate cell–cell and cell–matrix interactions, but may also provide answers to CVDs that arise from cells interacting with CMs. For instance, two examples of diseases that would benefit from such an approach would be Marfan syndrome, which leads to cardiovascular defects because of dysfunctional connective tissue (Pepe et al., 2016), and the hypoplastic left heart syndrome (HLHS) (Miao et al., 2020).

Looking at translational potential, the development of new drugs is a long and tedious process that aims at the identification of potential drug-induced adverse effects (e.g., cardiotoxicity) (Ovics et al., 2020). The implementation of hiPSCs in the drug development pipeline will therefore enable to assess patient-specific drug responses and to perform early drug de-risking, thus reducing the number of “bad” lead compounds candidates progressing from the pre-clinical to clinical trials (Figure 3).

Next, the pluripotency potential of hiPSCs could also be further combined to the genetic tool CRISPR-Cas9 aiming at the generation of isogenic cell lines for regenerative medicine. These isogenic hiPSCs can then be differentiated into the desired cell types and be used as a building block to create constructs for the replacement of damaged cardiovascular tissues (Figure 3). This process can be applied to personalized tissue regeneration by using a patient-specific cell for the production of autologous tissue constructs, thereby avoiding immunogenicity issues. Furthermore, the potential of CRISPR-Cas9 to generate HLA deficient hiPSCs would grant a universal cell source with reduced immunogenicity. This will eradicate the need for autologous hiPSCs for cardiac regeneration, thereby reducing time and cost constraints associated with patient-specific cells.

However, hiPSCs still present some concerns that need to be addressed before their safe and effective translation into the clinical setting will be possible. Studies showed that hiPSCs can be subject to chromosomal aberrations, which can either be inherited from the parental cells or arise from the cellular reprogramming or prolonged culture periods, finally impacting their differentiation potential and disease modeling (Mayshar et al., 2010; Yoshihara et al., 2017). Moreover, hiPSC-derived CMs are subject to specific limitations, such as the lack of heterogenous cell population after differentiation into CMs and lack of maturity (Gherghiceanu et al., 2011; Bedada et al., 2014; Veerman et al., 2015; Koivumäki et al., 2018). These aspects considerably limit the ability of hiPSCs-derived CMs to reliably mirror the complete phenotype of mature CMs. Furthermore, the immature phenotype reduces the ability of hiPSC-derived CMs to model CVDs that manifest at a later developmental stage.

TE strategies present also some limitations that need to be addressed before their broader clinical translation, such as scaffolds biocompatibility and mechanical properties, cell–cell interactions, cell–ECM interactions, and the vascularization potential. Nonetheless, the rapid evolution in hiPSC and CRISPR-Cas9 technologies combined to TE strategies carries an enormous potential to advance the field of regenerative cardiovascular research to the next level.

## Conclusion

The discovery of patient-specific hiPSCs has revolutionized the field of cardiovascular research. The differentiation potential of hiPSCs into CMs and their ability to retain the genetic background enable the generation of CVD models and investigate the underlying mechanisms responsible for pathological phenotype. On the other hand, advances in genome engineering promoted by the CRISPR-Cas9 technology enabled the generation of isogenic hiPSCs owing to specific genetic mutations but also the correction of single mutations involved in CVDs. Hence, hiPSC and CRISPR-Cas9 technologies are providing a novel treatment option for personalized medicine, and the potential combination of hiPSCs and CRISPR-Cas9 together with TE approaches could allow the generation of specific 3D disease models systems and various tissue-engineered constructs for cardiovascular regenerative purposes.

## Author Contributions

EKNG, SEM, and MYE conceptualized the manuscript. EKNG and SEM drafted and edited the manuscript. SEM, MM, BN, and MYE revised and/or edited the manuscript. Supervisory and administrative tasks were handled by SEM, MYE, and SPH. All authors contributed to the manuscript with critical input.

## Conflict of Interest

SPH is shareholder at Xeltis BV and LifeMatrix AG. MYE is a shareholder at LifeMatrix. BN is a AstraZeneca employee and shareholder and declares no competing interests. The remaining authors declare that the research was conducted in the absence of any commercial or financial relationships that could be construed as a potential conflict of interest.
